# Supplementary material for: A Likelihood Approach for Real-Time Calibration of Stochastic Compartmental Epidemic Models
Source: PLoS Comput Biol. 2017 Jan 17;13(1):e1005257. doi: 10.1371/journal.pcbi.1005257 (PMC5240920; doi:10.1371/journal.pcbi.1005257)
Supplement: S1 File — (TAR.GZ) [file pcbi.1005257.s014.tar.gz › HSPH_Online-SI-Revision/EnKF07/n100-mild/n100-mild_EnKF_table-4.pdf]

1	R0	{1.24125, 3.38982}	{2.26081, 5.79689}	{{0.0801808, 3.46807}, {3., 12.4888}}	{108.732, 71.0088}	inf	1007.	8215.2	{235.333, 4279.66}	738.469	0.781215	newly	2409.	5776.75	{216., 6342.}	170.442	0.500951	Reff	1.16056	0.484159	{0.0228732, 0.71335}	59.7886	ar	34926.	184359.	{166842., 177712.}	427.857
2	R0	{1.29554, 4.51878}	{2.44432, 6.70607}	{{0.880111, 0.657296}, {3., 19.272}}	{103.405, 134.392}	inf	1718.	5344.91	{875.905, 5933.75}	227.796	0.509786	newly	2989.	8666.95	{546., 13604.}	238.805	0.561353	Reff	1.16829	0.573241	{0.135887, 0.877919}	60.261	ar	42828.	184363.	{170374., 225894.}	330.473
3	R0	{1.2443, 3.64292}	{2.20823, 8.87918}	{{0.368114, 2.63213}, {3., 13.4935}}	{111.227, 157.584}	inf	1255.	4283.12	{397.981, 2704.99}	260.435	0.443016	newly	2853.	3488.04	{188., 4141.}	97.3649	0.409394	Reff	1.15569	0.406871	{0.0721819, 0.637119}	65.7985	ar	37087.	178464.	{169374., 187965.}	381.203
4	R0	{1.38465, 3.43331}	{2.17665, 4.11186}	{{1.07, 2.64879}, {3., 4.94115}}	{71.553, 30.7318}	inf	1365.	15617.4	{5056.84, 50940.1}	1046.43	0.914637	newly	3916.	18884.1	{5252., 56143.}	386.267	0.514062	Reff	1.30897	0.4305	{0.0518316, 1.04941}	69.1809	ar	49511.	167917.	{99595., 241804.}	239.15
5	R0	{1.25409, 4.1068}	{2.16383, 9.01663}	{{0.359471, 2.64621}, {3., 13.4232}}	{109.569, 136.598}	inf	1456.	4182.97	{422.047, 2735.95}	214.742	0.454899	newly	2839.	3428.9	{202., 4173.}	99.8739	0.498953	Reff	1.15606	0.402861	{0.0705626, 0.634369}	66.0911	ar	38571.	177825.	{168857., 188862.}	361.033
6	R0	{1.28821, 4.2016}	{2.59118, 5.73821}	{{1.62384, 3.478}, {3., 12.3767}}	{103.127, 54.483}	inf	1742.	7513.1	{282.759, 4286.39}	378.418	0.583392	newly	3382.	6785.66	{287., 7733.}	164.485	0.526299	Reff	1.16363	0.62612	{0.110575, 0.736802}	57.7582	ar	41468.	185902.	{165501., 228246.}	348.303
7	R0	{1.24369, 2.56007}	{2.04734, 4.11292}	{{0.0713874, 0.944703}, {3., 5.78968}}	{106.084, 93.4601}	inf	601.	5266.32	{735.996, 14296.}	779.195	0.722103	newly	2262.	6841.53	{585., 16564.}	244.42	0.452328	Reff	1.20376	0.33483	{0.0106179, 0.659998}	73.0614	ar	38603.	166415.	{156178., 200442.}	333.296
8	R0	{1.232, 3.53942}	{2.42142, 7.03525}	{{0.913465, 0.651787}, {3., 19.3567}}	{108.94, 173.659}	inf	1387.	5058.61	{752.001, 5826.83}	280.023	0.557592	newly	3108.	8151.22	{659., 11468.}	213.575	0.537256	Reff	1.11866	0.542747	{0.125302, 0.689979}	62.1584	ar	34555.	183891.	{168946., 226897.}	432.169
9	R0	{1.36262, 3.85631}	{2.4971, 4.52673}	{{1.99664, 1.10894}, {3., 7.42945}}	{85.3192, 61.1788}	inf	1592.	1995.73	{704.52, 2716.96}	83.3674	0.2549	newly	3831.	3507.07	{677., 6508.}	101.967	0.531989	Reff	1.27209	0.551788	{0.176031, 1.70273}	68.5318	ar	48410.	182278.	{166523., 227942.}	276.529
10	R0	{1.28702, 4.67968}	{2.72521, 4.47856}	{{2.19056, 1.27988}, {3., 6.27207}}	{111.745, 39.6866}	inf	1874.	3152.97	{839.351, 2632.22}	82.5705	0.148311	newly	3114.	6351.74	{1404., 4004.}	172.763	0.303813	Reff	1.13924	0.498966	{0.106868, 0.750898}	67.972	ar	41092.	184501.	{173559., 195331.}	348.996
11	R0	{1.35752, 3.47129}	{2.15515, 8.8975}	{{0.383804, 2.67318}, {3., 13.6209}}	{96.0197, 167.788}	inf	974.	4204.39	{127.912, 2771.45}	360.44	0.629306	newly	2552.	3422.12	{48., 4091.}	103.587	0.504404	Reff	1.29461	0.389882	{0.071958, 0.646258}	70.5646	ar	44669.	176824.	{168198., 176134.}	295.854
12	R0	{1.35929, 5.83647}	{2.72459, 4.36172}	{{2.17465, 1.29084}, {3., 6.27657}}	{100.442, 32.8084}	inf	2459.	3129.16	{797.983, 3839.55}	74.2677	0.20982	newly	3330.	6414.4	{1290., 6472.}	169.125	0.343008	Reff	1.20052	0.52702	{0.127754, 0.758886}	68.5703	ar	46715.	183769.	{169745., 196735.}	293.384
13	R0	{1.28019, 3.29908}	{2.49599, 6.5383}	{{1.44119, 3.30894}, {3., 10.1553}}	{94.9702, 98.8488}	inf	1185.	2201.94	{1465.48, 2718.13}	95.6926	0.265224	newly	3104.	3144.7	{719., 3627.}	106.173	0.526586	Reff	1.21037	0.469521	{0.0307558, 0.718034}	64.5026	ar	40941.	178828.	{168101., 201014.}	336.794
14	R0	{1.36661, 3.48318}	{1.9948, 4.13681}	{{0.0388443, 0.947914}, {3., 5.76942}}	{92.5736, 61.2416}	inf	1869.	5176.33	{981.935, 17422.3}	197.928	0.370991	newly	4569.	5917.16	{814., 16231.}	95.9624	0.38088	Reff	1.26507	0.267285	{0.00542511, 0.452859}	78.8719	ar	48672.	166697.	{158108., 204348.}	244.505
15	R0	{1.35865, 3.84123}	{2.16293, 8.6112}	{{0.372814, 2.66239}, {3., 13.5795}}	{95.4434, 141.337}	inf	1321.	5010.99	{375.619, 2792.4}	300.304	0.453263	newly	3065.	3883.02	{175., 4266.}	101.663	0.419993	Reff	1.28034	0.411516	{0.0730782, 0.638269}	68.5328	ar	48598.	179175.	{169183., 196231.}	268.688
16	R0	{1.22879, 3.98329}	{2.72976, 4.41697}	{{2.17133, 1.35568}, {3., 6.25962}}	{122.15, 47.4025}	inf	1186.	2983.25	{567.818, 3342.03}	173.595	0.361792	newly	2363.	6025.09	{927., 5157.}	212.431	0.319824	Reff	1.12545	0.462037	{0.125849, 0.637023}	71.1439	ar	34405.	184014.	{172724., 195638.}	434.846
17	R0	{1.34925, 3.64524}	{2.5194, 4.43303}	{{1.9998, 1.19846}, {3., 7.44388}}	{88.1926, 64.2864}	inf	1082.	1955.54	{715.349, 2773.74}	103.005	0.210735	newly	2774.	3465.64	{661., 6297.}	117.686	0.479828	Reff	1.28476	0.576485	{0.17067, 1.90178}	68.044	ar	46871.	182721.	{166553., 247739.}	289.838
18	R0	{1.33054, 2.25275}	{2.49879, 7.30268}	{{2.16707, 4.26004}, {3., 14.6893}}	{88.6834, 225.709}	inf	387.	22478.1	{5934.1, 45066.5}	5708.3	1.61531	newly	1979.	24971.3	{5794., 63060.}	1163.48	0.924319	Reff	1.30961	1.04927	{0.323977, 2.18439}	52.5834	ar	44336.	156392.	{93357., 224651.}	253.121
19	R0	{1.28394, 3.69859}	{2.3909, 6.95414}	{{0.929998, 0.618209}, {3., 19.3225}}	{100.527, 167.864}	inf	1262.	5021.57	{500.877, 5947.51}	316.523	0.624831	newly	2837.	8435.19	{277., 13109.}	250.252	0.622173	Reff	1.18844	0.54997	{0.186563, 0.672354}	61.2966	ar	39558.	182351.	{168406., 223828.}	360.97
20	R0	{1.2883, 2.26493}	{2.55513, 6.98867}	{{2.16296, 4.25957}, {3., 10.6116}}	{100.045, 208.56}	inf	248.	21254.4	{6532.97, 37190.2}	8470.32	1.79139	newly	1268.	23270.4	{6122., 69758.}	1737.21	1.0948	Reff	1.27318	0.953349	{0.325812, 2.13409}	51.9979	ar	40768.	147609.	{84102., 227605.}	262.694
21	R0	{1.34098, 2.78621}	{2.51072, 7.59687}	{{2.17033, 4.14508}, {3., 14.7433}}	{90.1358, 174.11}	inf	675.	21182.3	{8055.59, 34951.5}	3038.11	1.40284	newly	2390.	22025.6	{6472., 60779.}	822.547	0.819317	Reff	1.30397	0.915459	{0.238595, 2.1873}	53.8681	ar	44605.	149887.	{95852., 222432.}	236.428
22	R0	{1.33783, 5.95911}	{2.74612, 4.40044}	{{2.2291, 1.27287}, {3., 6.27171}}	{105.266, 31.2969}	inf	2154.	4209.9	{725.546, 6863.31}	119.958	0.186961	newly	2912.	8533.57	{1099., 6794.}	256.183	0.328171	Reff	1.18802	0.527368	{0.138138, 0.781872}	68.2322	ar	45418.	184430.	{170683., 193421.}	306.072
23	R0	{1.33359, 3.3069}	{2.48903, 6.65829}	{{1.44698, 3.32901}, {3., 10.1707}}	{86.6414, 101.98}	inf	965.	2984.14	{1471.88, 4547.1}	210.278	0.29386	newly	2751.	3961.88	{751., 3731.}	135.581	0.415146	Reff	1.27359	0.508513	{0.0267989, 0.983891}	64.668	ar	46245.	181742.	{169471., 216411.}	292.998
24	R0	{1.30588, 2.56123}	{2.4577, 4.65472}	{{1.77902, 1.08201}, {3., 12.7081}}	{88.7245, 127.958}	inf	683.	20944.2	{4541.36, 54165.8}	2967.66	1.31357	newly	2554.	26296.6	{2341., 71472.}	936.023	0.820336	Reff	1.26499	0.638414	{0.153663, 1.35903}	54.8151	ar	42383.	153902.	{93864., 246930.}	263.122
25	R0	{1.29813, 4.51849}	{2.7196, 4.41551}	{{2.17915, 1.34739}, {3., 6.26223}}	{109.501, 41.6423}	inf	1351.	2854.96	{597.082, 3649.93}	141.381	0.358965	newly	2397.	5847.11	{972., 6250.}	206.872	0.357561	Reff	1.19921	0.488264	{0.131939, 0.663893}	71.8508	ar	43371.	185069.	{172456., 222073.}	326.712
26	R0	{1.33231, 3.99953}	{2.23003, 5.68959}	{{0.0710209, 3.46831}, {3., 12.4556}}	{96.1317, 56.0698}	inf	1549.	7948.06	{80.9613, 4425.2}	448.529	0.647279	newly	3228.	6299.81	{89., 6494.}	143.072	0.461324	Reff	1.2303	0.461464	{0.0153019, 0.713869}	64.6712	ar	44733.	183155.	{165893., 177620.}	309.44
27	R0	{1.30555, 4.61445}	{2.72668, 4.39126}	{{2.18827, 1.3831}, {3., 6.28787}}	{108.853, 41.1016}	inf	1634.	2888.17	{606.781, 3693.88}	109.551	0.321454	newly	2878.	5888.29	{918., 6281.}	179.644	0.397222	Reff	1.20568	0.491947	{0.124877, 0.693097}	72.0231	ar	42624.	185239.	{172879., 197959.}	334.589
28	R0	{1.26819, 5.34969}	{2.44921, 6.74061}	{{0.90906, 0.623126}, {3., 19.3142}}	{106.137, 120.148}	inf	1779.	5276.5	{1650.16, 5952.23}	212.322	0.515551	newly	2612.	8523.87	{554., 12408.}	271.098	0.607841	Reff	1.1566	0.562418	{0.13995, 0.692188}	61.1192	ar	38946.	183853.	{170527., 225914.}	372.07
29	R0	{1.19925, 3.00394}	{2.55832, 13.0521}	{{1.31028, 4.90616}, {3., 18.63}}	{113.327, 334.5}	inf	770.	2610.75	{788.646, 2723.}	251.627	0.438071	newly	2181.	2536.67	{312., 2344.}	125.776	0.523733	Reff	1.12966	0.546309	{0.225975, 1.22959}	60.9905	ar	31727.	178172.	{167680., 203410.}	461.58
30	R0	{1.35749, 3.75483}	{2.4966, 6.53729}	{{1.44214, 3.31057}, {3., 10.1482}}	{83.9132, 82.2174}	inf	1440.	2260.49	{1470.83, 2848.3}	61.2813	0.123629	newly	3590.	3178.98	{736., 3663.}	101.589	0.508124	Reff	1.28281	0.469656	{0.0284372, 0.726243}	65.6024	ar	48244.	179010.	{168215., 201329.}	271.052
31	R0	{1.27131, 4.2161}	{2.25046, 5.78142}	{{0.102849, 3.46613}, {3., 12.4939}}	{103.142, 55.6269}	inf	1285.	8801.98	{180.68, 4280.2}	613.034	0.689475	newly	2456.	6884.44	{167., 6304.}	214.914	0.48412	Reff	1.18819	0.496088	{0.0327976, 0.709497}	60.0769	ar	38299.	185665.	{166658., 177625.}	384.778
32	R0	{1.31827, 6.18119}	{2.72445, 4.47727}	{{2.18573, 1.2823}, {3., 6.26773}}	{106.669, 29.0222}	inf	2473.	3118.88	{750.449, 3310.6}	74.1747	0.209181	newly	3092.	6284.49	{1150., 5068.}	178.924	0.337594	Reff	1.16063	0.47967	{0.105588, 0.633328}	70.0158	ar	45262.	185301.	{174435., 196904.}	309.396
33	R0	{1.20398, 1.35167}	{2.62741, 7.1932}	{{2.16143, 4.28873}, {3., 14.7037}}	{118.863, 432.171}	inf	104.	22429.5	{5928.51, 42653.}	21466.9	2.19286	newly	1068.	22049.8	{5792., 45677.}	1964.59	1.16023	Reff	1.19578	0.916575	{0.320886, 2.18599}	49.2145	ar	33329.	150481.	{80145., 222260.}	351.583
34	R0	{1.32776, 3.146}	{2.50113, 4.38291}	{{1.99855, 1.13801}, {3., 7.44169}}	{90.0981, 81.0293}	inf	1228.	1930.88	{731.732, 2315.36}	86.9659	0.182327	newly	3687.	3419.18	{695., 4637.}	98.1915	0.489874	Reff	1.25318	0.586933	{0.17792, 1.99853}	67.9847	ar	44872.	183012.	{166358., 247050.}	307.854
35	R0	{1.279, 1.11336}	{2.48405, 7.56207}	{{2.13096, 4.31023}, {3., 14.7082}}	{94.6419, 579.211}	inf	39.	22750.7	{5243.37, 44582.1}	58235.1	2.6037	newly	625.	26709.5	{3928., 71490.}	4175.36	1.41042	Reff	1.27363	1.06503	{0.330697, 2.1881}	50.7016	ar	41033.	158725.	{101370., 232948.}	287.263
36	R0	{1.20029, 1.94717}	{2.50545, 7.00738}	{{2.16366, 4.23951}, {3., 14.686}}	{109.063, 261.364}	inf	272.	22774.6	{6474.16, 42877.8}	8273.	1.78889	newly	1470.	26356.9	{6220., 69800.}	1693.86	1.08341	Reff	1.18231	1.06612	{0.327441, 2.18729}	53.4134	ar	33234.	159560.	{97682., 233061.}	380.112
37	R0	{1.33216, 3.69685}	{2.56946, 12.3785}	{{1.28008, 4.88636}, {3., 18.5793}}	{94.0157, 234.839}	inf	1487.	2705.15	{635.175, 2579.84}	101.781	0.246968	newly	3432.	2634.86	{472., 1787.}	108.224	0.584926	Reff	1.23359	0.554191	{0.241761, 0.922343}	61.4214	ar	45235.	177277.	{166865., 199828.}	291.902
38	R0	{1.25434, 2.04314}	{2.53371, 6.81551}	{{2.14178, 4.25065}, {3., 14.7434}}	{103.37, 233.58}	inf	320.	18872.9	{6469.74, 32646.}	5797.77	1.66751	newly	1505.	23123.9	{5766., 70160.}	1437.79	1.03038	Reff	1.23694	1.06307	{0.381722, 2.18878}	49.5712	ar	38662.	154352.	{85024., 228904.}	299.297
39	R0	{1.27466, 3.0741}	{1.98892, 4.16494}	{{0.045484, 0.953857}, {3., 5.74805}}	{102.29, 72.7946}	inf	1446.	5089.01	{862.765, 14574.1}	267.673	0.429929	newly	3912.	5782.96	{681., 16274.}	108.261	0.385064	Reff	1.18848	0.274917	{0.00638242, 0.454237}	76.8682	ar	40181.	165324.	{156358., 196252.}	312.302
40	R0	{1.33045, 2.68154}	{2.45854, 4.55725}	{{1.80578, 1.13055}, {3., 12.7037}}	{85.3447, 118.299}	inf	888.	22429.3	{4452.16, 58313.4}	2427.05	1.22361	newly	3300.	27133.8	{2425., 72407.}	731.715	0.749486	Reff	1.28496	0.600685	{0.126159, 1.04141}	56.3272	ar	45951.	152531.	{94690., 245868.}	231.943
41	R0	{1.37003, 2.83984}	{2.50779, 7.18181}	{{2.16892, 4.22386}, {3., 14.693}}	{84.052, 155.921}	inf	521.	22622.9	{5928.21, 45323.}	4242.21	1.49211	newly	2106.	25230.	{5844., 63095.}	1100.4	0.909881	Reff	1.34277	1.04165	{0.248379, 2.18559}	53.0319	ar	48926.	156081.	{95664., 224548.}	219.504
42	R0	{1.3003, 2.94885}	{2.10538, 4.13909}	{{0.0639668, 0.943358}, {3., 5.79472}}	{101.843, 78.5994}	inf	940.	4468.52	{678.431, 8210.25}	385.471	0.519279	newly	2995.	5491.73	{581., 9451.}	139.706	0.414245	Reff	1.24816	0.307853	{0.00959564, 0.457673}	75.3915	ar	44237.	161278.	{147681., 189089.}	268.169
43	R0	{1.33756, 2.92653}	{2.50761, 7.20099}	{{2.16764, 4.23174}, {3., 14.6902}}	{88.3636, 149.285}	inf	626.	22445.8	{5918.17, 45077.}	3485.59	1.40514	newly	1978.	25075.1	{5789., 63560.}	1169.6	0.928373	Reff	1.31066	1.04989	{0.325941, 2.18542}	52.4815	ar	47342.	156564.	{93656., 224567.}	231.167
44	R0	{1.35801, 2.92276}	{2.45441, 4.7965}	{{1.76752, 1.16996}, {3., 12.7476}}	{81.345, 110.074}	inf	645.	20631.4	{4172.68, 54081.}	3098.86	1.32381	newly	2455.	25970.2	{2360., 72577.}	963.441	0.820192	Reff	1.32668	0.655333	{0.167274, 1.36199}	54.7327	ar	47940.	155547.	{97606., 247533.}	224.463
45	R0	{1.31342, 4.02649}	{2.50048, 6.61326}	{{1.43997, 3.31595}, {3., 10.0937}}	{90.3795, 75.9334}	inf	1370.	2891.93	{1447.2, 4470.03}	118.568	0.221955	newly	2863.	3884.64	{707., 3676.}	134.291	0.520345	Reff	1.22662	0.504568	{0.0293842, 0.979201}	64.2855	ar	42995.	181729.	{168319., 215799.}	322.674
46	R0	{1.37919, 3.75889}	{2.52984, 4.57937}	{{2.002, 1.20554}, {3., 7.4831}}	{84.6305, 63.7008}	inf	1025.	2338.21	{731.523, 2755.47}	142.589	0.194948	newly	2667.	4064.33	{681., 12114.}	141.521	0.459211	Reff	1.32725	0.614847	{0.179564, 2.00198}	66.8732	ar	49157.	185043.	{166378., 247557.}	276.432
47	R0	{1.37458, 3.56117}	{2.54229, 4.47141}	{{2.00042, 1.16393}, {3., 7.47382}}	{86.5376, 69.5947}	inf	1179.	2007.99	{723.109, 2772.62}	96.8764	0.190053	newly	3193.	3448.49	{672., 6591.}	109.349	0.479767	Reff	1.31164	0.524407	{0.171075, 1.04247}	69.3577	ar	48478.	180894.	{166415., 219432.}	273.146
48	R0	{1.33393, 3.78296}	{2.3923, 7.00393}	{{0.935793, 0.586349}, {3., 19.4212}}	{94.8444, 164.478}	inf	1368.	5127.43	{801.662, 5977.63}	290.747	0.605809	newly	3101.	8235.72	{252., 12303.}	217.289	0.593876	Reff	1.2473	0.517055	{0.14383, 0.676182}	65.1609	ar	44885.	182514.	{170463., 224122.}	306.627
49	R0	{1.24918, 2.39046}	{2.11697, 4.19575}	{{0.0484942, 0.952974}, {3., 5.7874}}	{107.919, 106.782}	inf	662.	4833.84	{787.099, 8164.82}	633.114	0.661624	newly	2523.	6381.41	{610., 9699.}	199.254	0.415378	Reff	1.19869	0.327889	{0.00734123, 0.540867}	73.5723	ar	35969.	163217.	{153042., 188581.}	354.507
50	R0	{1.28281, 2.3032}	{2.17591, 4.11372}	{{1.05711, 2.64798}, {3., 4.97353}}	{80.0633, 78.6089}	inf	746.	15361.	{5061.2, 51035.9}	1960.96	1.16654	newly	2869.	18686.8	{5291., 56232.}	554.822	0.643775	Reff	1.23601	0.441666	{0.0489187, 1.0209}	66.8372	ar	40223.	160497.	{95357., 238983.}	300.783
